# Reprogramming Tumor Associated Macrophage Phenotype by a Polysaccharide from *Ilex asprella* for Sarcoma Immunotherapy

**DOI:** 10.3390/ijms19123816

**Published:** 2018-11-30

**Authors:** Qiu Li, Zhihui Hao, Yeting Hong, Wei He, Wenwen Zhao

**Affiliations:** 1 Agricultural Bio-Pharmaceutical Laboratory, College of Chemistry and Pharmaceutical Sciences, Qingdao Agricultural University, Qingdao 266109, China; abplab@126.com; 2 College of Laboratory Medicine, Hangzhou Medical College, Binwen Road, Hangzhou 310053, China; hongyeting123@163.com; 3 Department of Immunology, School of Basic Medical Sciences, Anhui Medical University, Hefei 230032, China; 4 Department of Pharmacology, College of Basic Medicine, Qingdao University, Qingdao 266071, China; wenwenzhao0313@163.com

**Keywords:** IAPS-2 polysaccharide, repolarizing TAMs, cancer immunotherapy

## Abstract

We report here the discovery of an acidic polysaccharide, namely IAPS-2, from the root of *Ilex asprella*, with anti-tumor activity via a repolarizing tumor associated macrophages (TAMs) phenotype. We obtained IAPS-2 polysaccharide from this herb based on acidity and found that IAPS-2 expressed the activity of promoting the secretion of anti-tumor cytokines in macrophages. Furthermore, we evaluated its anti-tumor effect on TAM cells, through the activation of nuclear factor-κB (NF-κB) and signal transducer and activator of transcription (STAT) signaling. In particular, in the tumor murine model, IAPS-2 demonstrated that it could significantly inhibit the growth of tumors via modulating the function of TAMs and increase the animal survival rate. In summary, IAPS-2, with a clearly illustrated chemical composition, potent anti-tumor activity, and a solid mechanism of action, may be developed into a valuable therapeutic tool for cancer immunotherapy.

## 1. Introduction

The therapeutic tools that modulate the immune system hold great promise for cancer immunotherapy [1,2]. At present, the main strategies of cancer immunotherapy are the administration of protein drugs or cellular therapy [3,4,5], but they both have the limitations of instability, technical complexity and high cost. Meanwhile, the active polysaccharides derived from diverse natural origins are commonly found to produce various immunomodulatory effects in vitro and in vivo [6,7,8]. More and more studies indicate that polysaccharides have an evident advantage of targeting and modulating tumor immunity [9,10], which can kill tumor cells, and not induce the acquired resistance. Therefore, developing and exploring active polysaccharides for modulating a tumor immune microenvironment may be of great value.

Tumor associated macrophages (TAMs) are one type of main immune cells which have demonstrated the effects of driving the initiation, proliferation, metastasis and angiogenesis of diverse tumors [11,12,13]. They have become a key barrier for cancer immunotherapy [14,15,16]. Numerous investigations have indicated that reclaiming TAMs into M1 type was an effective strategy for treating cancers [17,18]. As such, searching for active polysaccharides that are capable of modulating TAM type and function, is a promising prospect for cancer immunotherapy.

The root of *Ilex asprella*, a Chinese medicinal herb, is commonly used in the south of China [19,20]. In our previous study, we reported an active polysaccharide, namely IAPS-2, that demonstrated an immune-potentiator, which can increase the secretion of major inflammatory cytokines [19]. Accordingly, we were inspired to question whether IAPS-2 could modulate a TAM phenotype, and further re-educate the tumor microenvironment for tumor immunotherapy. Therefore, in this study, we first tested the activity of IAPS-2 and investigated its effect on the phenotype change of TAMs. Then, we observed its effect on the correlative signal pathways and identified the target proteins. Furthermore, we evaluated its anti-tumor activity in S180 (a syngeneic sarcoma) tumor-bearing mice. All the results suggested that IAPS-2 had a potent effect on remodeling the TAM type and may be developed into a therapeutic tool for cancer immunotherapy.

## 2. Results and Discussion

### 2.1. Effect of IAPS-2 on Macrophages and TAMs

**Figure 1:**
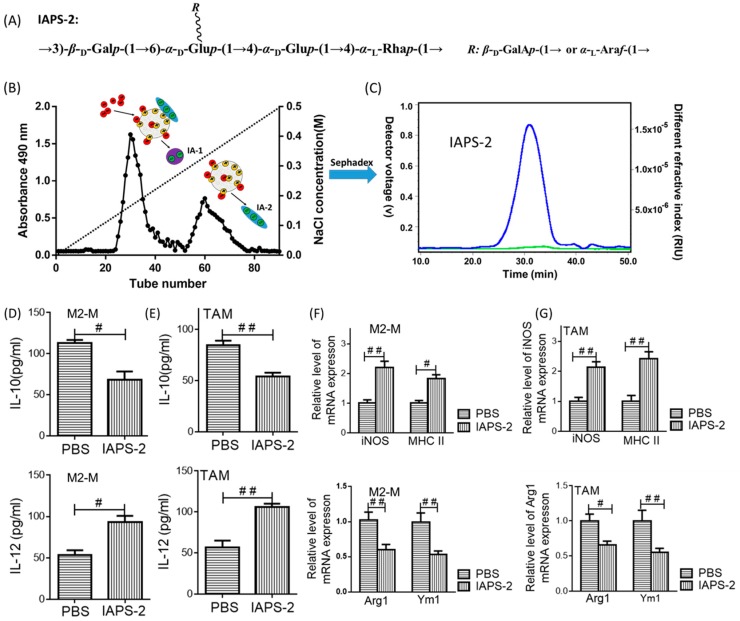
IAPS-2 promoted M2-macrophage toward M1-type polarization. M2-M and tumor associated macrophages (TAMs) were treated with IAPS-2 (50 μg/mL) for 24 h. (**A**) The chemical structure of IAPS-2; (**B**) the scheme to isolate different IA fractions based on acidity (Red, NaCl solution, purple, fraction IA-1, blue, fraction IA-2); (**C**) the purification statement of IAPS-2, which has been presented in our previously published paper; the concentration of IL-10, IL-12 in the supernatants from M2-M and TAMs (**D**,**E**) were tested by ELISA and the gene expression of M1-marker (*iNOS and MHC II*) and M2-marker (*Arg1 and Ym1*) in both cells were also examined by Q-PCR (**F**,**G**). The data are representative of three independent experiments and expressed as the means ± SD. ^#^ *p* < 0.05 or ^##^ *p* < 0.01 compared with the phosphate-buffered saline (PBS) group.

Following our published in-house protocol [19,21], we isolated polysaccharides from *Ilex asprella* using ion-exchange chromatography, which comprise two main fractions, namely IA-1, IA-2 (Figure 1B). After purifying them by sephadex gel, we obtained the homogeneous polysaccharide, namely IAPS-2, and fully characterized the chemical structure of IAPS-2 (Figure 1A,C). Moreover, we preliminary found that IAPS-2 could affect the secretion of major inflammatory cytokines [19]. Therefore, we supposed that IAPS-2 possessed a potent capability and that it was therefore worthwhile to study it in depth. In this study, we observed the effect of IAPS-2 on M2-type macrophages and TAMs in vitro. We chose to treat IL-4-cultured RAW 264.7 macrophages (M2-M) and TAMs with IAPS-2 and examined the phenotype change of the cells. First, the levels of two cytokines (IL-10 and IL-12) from the cell supernatant were measured with the commercial kits. The results indicated that IAPS-2 could promote the secretion of IL-12 and decrease the level of IL-10, compared with the control group (*p* < 0.05 or *p* < 0.01) (Figure 1D,E). Second, the transcriptional levels of four genes (*Arg1, Ym1, iNOS and MHC II*) were measured. Interestingly, IAPS-2 exhibited a unique function, which evidently induced up-regulating the *iNOS, MHC II* levels, and down-regulating the *Arg1, Ym1* levels, compared with the control group (*p* < 0.05 or *p* < 0.01) (Figure 1F,G). Both the M2-M (polarized murine RAW 264.7 cells) and TAMs from S180 tumor tissues treated with IAPS-2 exhibited the same transformation. Taken together, the above outcomes demonstrated that IAPS-2 may have an anti-tumor effect.

### 2.2. The Change of Signal Transduction Regulated by IAPS-2 Polysaccharide In Vitro

**Figure 2:**
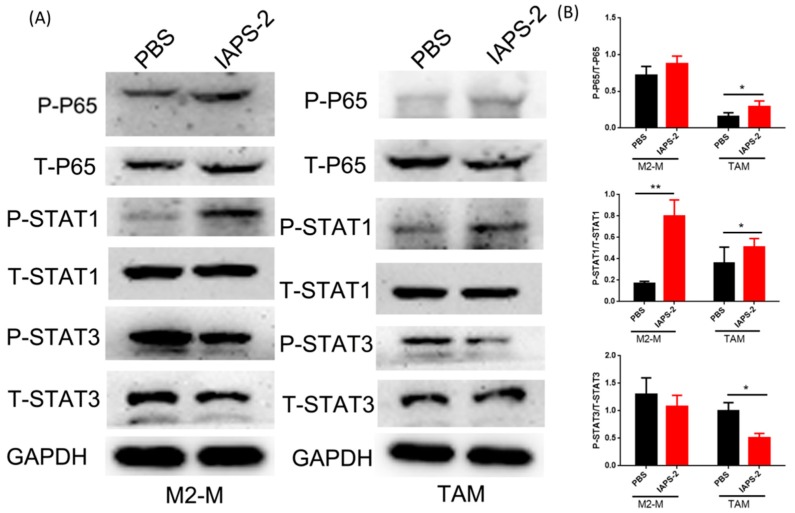
IAPS-2 induced repolarization of M2-type macrophage in vitro via activating NF-κB and STAT1 signaling and inhibiting STAT3 signaling. M2-M and TAMs were incubated with 50 μg/mL IAPS-2 for 24 h, (**A**) the expression of phosphorylated P65 (p-P65), total P65, phosphorylated STAT1 (p-STAT2), total STAT1 (T-STAT1), phosphorylated STAT3 (p-STAT3) and total STAT3 (T-STAT3) in both cells were evaluated by Western blot; (**B**) grey analysis of Western blotting. The data are representative of three independent experiments. The values are expressed as the means ± SD. * *p* < 0.05, ** *p* < 0.01.

We preliminarily identified the effect of IAPS-2 on macrophages. To further illustrate the underlying mechanism, we set out to test the regulation of IAPS-2 to the correlative signal pathways. In comparison, NF-κB, STAT1 signalling is substantial for macrophage polarization, which plays a vital role in this process. The up-regulating p-P65 and p-STAT1 levels are often found in M1 macrophages. In contrast, STAT3, a member of the STAT family of transcription factors, exerts essential functions in development and tissue homeostasis. Many studies reported that the activation of STAT3 could transfer macrophages into M2 type. In our study, we performed the WB assay to illustrate the influence of IAPS-2 on the corresponding signalling. The results confirmed that IAPS-2 could inhibit the phosphorylation of STAT3 in M2-M (polarized RAW cells) and TAMs from S180 tumor tissues. In addition, IAPS-2 could evidently enhance STAT1 phosphorylation and slightly promote P65 phosphorylation (Figure 2). Together, the results suggested that IAPS-2 is prone to enhancing M1 type differentiation in macrophages and TAMs via NF-κB, STAT signalling in vitro.

### 2.3. Effect of TAMs Treated with IAPS-2 on Tumors in Mice

**Figure 3:**
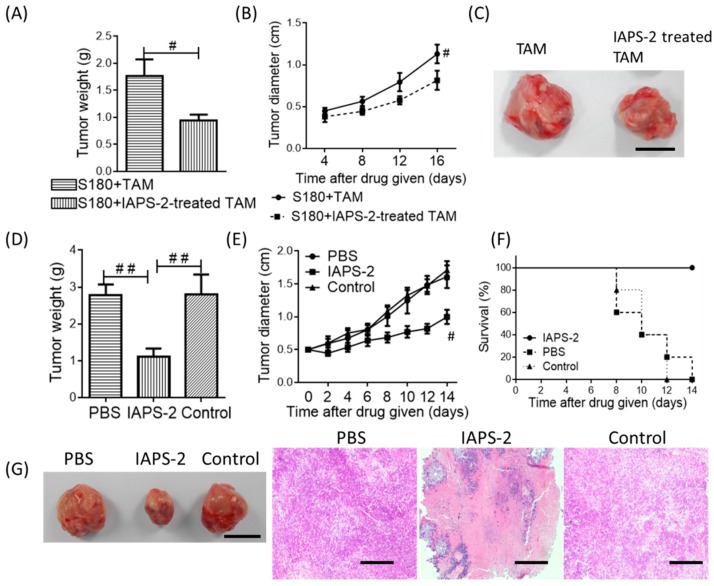
IAPS-2 treatment markedly retarded tumor growth. Mixed tumor cells with TAMs incubated with or without IAPS-2 and then these cells were injected into mice. Mean tumor weights (**A**), tumor growth curves (**B**) after mixed cells were injected into mice for 16 days. Representative images of tumor harvested from mice in each treatment cohort (**C**), scale bar, 1 cm. Mean tumor weights (**D**), tumor growth curves (**E**), and survival rate of tumor-bearing mice (# vs PBS group) (**F**) from mice receiving intratumoral injections of IAPS-2. Representative images of tumor (**G**, scale bar, 1 cm) and tumor sections examined by H&E staining (scale bar, 100 μm) from mice treated with 50 mg/kg IAPS-2. *n* = 6 mice/group. The values are expressed as the means ± SD. ^#^ *p* < 0.05, ^##^ *p* < 0.01.

To further determine whether the influence of IAPS-2 on TAMs leads to the anti-tumor effect, we mixed tumor cells with TAMs treated with or without IAPS-2 and then the mixture was administered to mice. The results suggested that in the mice injected with the cell-mixture pre-treated with IAPS-2 the tumor could significantly decrease in weight and size, and the tumor growth was also inhibited, compared with the groups treated with cell-mixture alone (Figure 3A–C). The obvious results indicated that IAPS-2 exerted the anti-tumor effect via the modulation of TAMs, and it could regulate the secretion of anti-tumor cytokines in tumors.

### 2.4. The Anti-Tumur Effect of IAPS-2 in Mice

#### 2.4.1. Effect of IAPS-2 on Tumor Growth

Accordingly, TAMs are the main immunosuppressive cells in tumor microenvironment and contribute cells-modulated immunosuppression in tumor immune escape. After having confirmed the polarization effect of IAPS-2 on TAMs from S180 tumor tissues in vitro, we set out to observe whether IAPS-2 can prevent the immunosuppressive function in tumors and ignite the anti-tumor effect of the immune system in tumor-bearing mice. The results indicated that the administration of IAPS-2 could significantly decrease the tumor weight and size (Figure 3D,E). In particular, IAPS-2 could effectively prolong the survival of the S180 tumor-bearing mice, compared with the other groups (Figure 3F). Furthermore, the mice in IAPS-2 group survived the whole test process. Additionally, the morphology of tumors was displayed in Figure 3G, which also demonstrated the anti-tumor effect of IAPS-2.

#### 2.4.2. The Histological Analysis

We further performed a histological analysis. H&E staining was used for the morphometric analysis of tumor tissue in mice. The results in Figure 3G showed that IAPS-2 could evidently induce tumor necrosis and inhibit tumor growth. Compared with the other control groups, its anti-tumor effect was superior, such as the decrease of tumor nuclei density.

### 2.5. Effect of IAPS-2 on TAMs in Tumor-Bearing Mice

**Figure 4:**
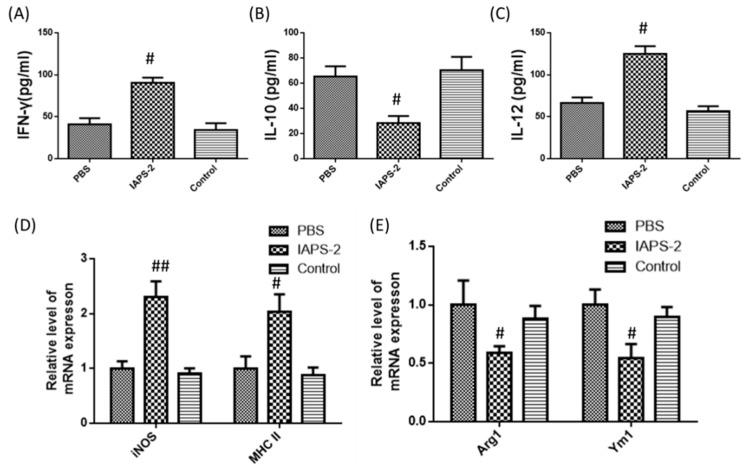
IAPS-2 altered TAMs phenotype and restored immunosurveillance against a tumor in vivo. TAMs purified from each treatment cohort were cultured for 24 h. The expression of (**A**–**C**) IFN-γ, IL-12, IL-10, and (**D**,**E**) iNOS, MHC II, Arg1 and Ym1 were examined by ELISA or q-PCR. The values are expressed as the means ± SD (*n* = 6 mice/group) vs. PBS group, ^#^ *p* < 0.05, ^##^ *p* < 0.01.

**Figure 5:**
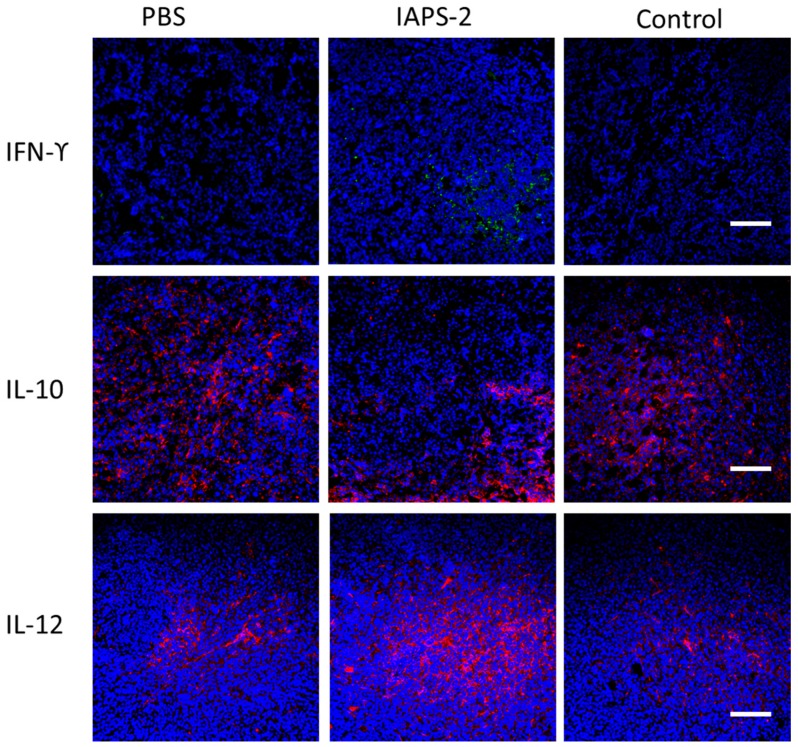
The immunofluorescence of S180 tumor sections was performed to test IL-12 and IL-10 secretion in tumor tissues. Red, IL-10 and IL-12; blue, 4′,6-diamidino-2-phenylindole (DAPI) nuclear staining, scale bar, 100 μm, and the levels of IFN-γ in tumor tissue were examined by immunofluorescent staining. Green, IFN-γ; blue, DAPI nuclear staining, scale bar, 100 μm.

Accordingly, we confirmed that IAPS-2 could regulate the function of TAMs in vitro. In particular, the modulating effect of IAPS-2 on TAMs for cancer immunotherapy in vivo was also demonstrated. Therefore, we set out to observe the phenotype change of TAMs isolated from tumors treated with IAPS-2. The results in Figure 4 suggested that the administration of IAPS-2 could promote the secretion of IL-12 and decrease the level of IL-10 in mice (Figure 4B,C). Meanwhile, the administration of IAPS-2 could increase the expression of genes associated with M1 type (*NOS2 and MHC II*) and down-regulate the expression of Arg1 and Ym1 commonly reckoned as M2 markers (Figure 4D,E). Meanwhile, the immunofluorescence staining of IL-10 and IL-12 in the tumor tissues treated with IAPS-2 was investigated and the graphic results also indicated the same trend, up-regulating level of IL-12 and down-regulating level of IL-10 (Figure 5). In addition, the expression of INF-γ, the marker of Th1 immune response, was significantly enhanced by the IAPS-2 treatment (Figure 4A and Figure 5) and suggested that IAPS-2 can eliminate an immune suppression and promote a Th-1-mediated immune response against tumors by switching to the TAMs phenotype. In this study, the direct administration of IAPS-2 was performed in S180 tumor-bearing mice. The results suggested that IAPS-2 had an obvious anti-tumor effect, which is attributed to the modulating effect on TAMs. As we know, TAMs are the main types of immunosuppressive cells in a tumor microenvironment, organizing the immune escape of tumors [22], which was a key blocker for cancer immunotherapy [23]. Indeed, high TAMs invasion has been involved in poor prognosis in several cancers, such as breast cancer, prostate carcinoma, colon cancer, and so on [24,25]. Macrophages are highly plastic cells and can be divided into a classical (M1) or an alternative (M2) phenotype [26,27]. M1-type macrophages exhibit tumoricidal activity and promote T lymphocytes-medicated tumor immunity by secreting IL-12 and nitric oxide (NO) [28]; whereas M2-polarized macrophages play an important role in tissue repair and enhance Th2 immune responses through the expression of IL-10 and arginase [29,30]. TAMs mostly show an M2-like phenotype, can affect tumor cell growth, metastasis, extracellular matrix remodeling, and so on [14,28]. Therefore, recent research has demonstrated that repolarizing TAMs into M1 type were effective in treating cancers. Similar to previous studies, we found that IAPS-2 can re-educate TAMs into a M1 phenotype to restore the local immune surveillance in the tumor microenvironment against tumor. In our study, we selected a syngeneic sarcoma mouse model (S180) for a series of studies. Although there are some differences between the different tumor entities, the S180 tumor model is thoroughly representative. Moreover, TAMs are the main immunosuppressive cells that exist in most tumor tissues. IAPS-2 can reprogram TAMs type and then show an anti-tumor effect. This suggests that the effect of IAPS-2 may be generalized from sarcoma to other tumor types.

### 2.6. Effect of IAPS-2 on TAMs Induced Angiogenesis in Tumor-Bearing Mice

**Figure 6:**
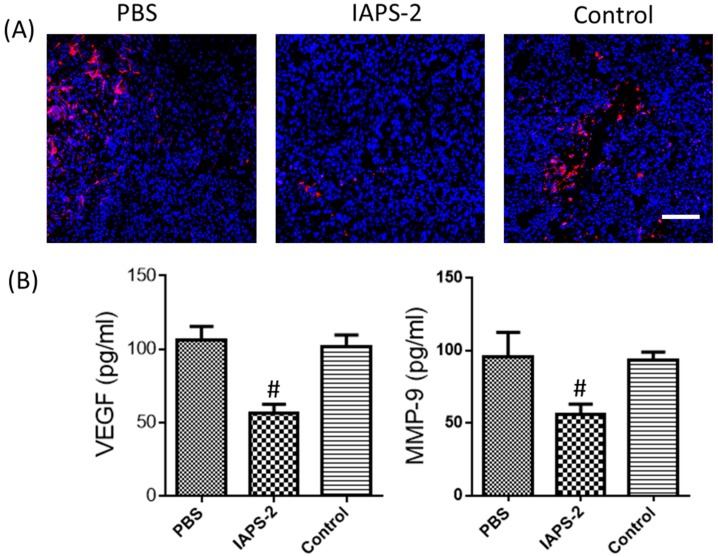
Repolarizing TAMs by IAPS-2 reduced S180 tumor angiogenesis. (**A**) Immunofluorescent staining for CD31 determines the amount of angiogenesis in tumors from each treatment cohort. Red, CD31; blue, DAPI nuclear staining, scale bar, 100 μm. *n* = 6 mice/group. (**B**) The expression of VEGF and MMP-9 in tumor tissues from each treatment cohort was examined by ELISA. The values are expressed as the means ± SD. ^#^ *p* < 0.05.

After we found that IAPS-2 could alter the type of macrophages and show an anti-tumor effect via regulating the function of TAMs, we further started to observe the effect of IAPS-2 on angiogenesis in tumors, which visually presented the growth of tumors. The analysis of the angiogenesis showed that IAPS-2 evidently reduced the concentration of MMP-9 and VEGF in S180 tumor tissues (Figure 6B). In particular, the immunofluorescence analysis of CD31 (an endothelial marker) significantly demonstrated that mice treated with IAPS-2 have a lower density of blood vessels (Figure 6A). Taken together, these analyses suggested that IAPS-2 displayed potent ability of anti-angiogenesis and can treat tumors.

## 3. Materials and Methods

### 3.1. Materials and Reagents

The root of *Ilex asprella* was obtained from Qingping herbal market and was identified by Guang-Xiong Zhou (Jinan University). Dulbecco’s modified Eagle’s medium (DMEM), penicillin/streptomycin antibiotic mixture and fetal bovine serum (FBS) were purchased from Life Technologies (Grand Island, New York, NY, USA). Other chemical reagents were purchased from Sangon Biotech (Shanghai, China). Murine RAW 264.7 cells, purchased from the American Type Culture Collection, were cultured in DMEM with 10% FBS, and incubated in 5% CO_2_ at 37 °C. The cells were seeded into 6-well plates in DMEM medium, supplemented with 10% FBS in 5% CO_2_ at 37 °C. Tumor-associated macrophages (TAMs) from S180 tumor tissues (a syngeneic sarcoma) were obtained by using a published method [31]. TAMs were cultured under the same condition with the above macrophages.

### 3.2. Preparation of IAPS-2 Polysaccharide

We prepared IAPS-2 polysaccharide according to our published in-house protocol [21]. Briefly, the air-dried *I. asprella* was first de-fatted using 95% ethanol. Then the dried residue was extracted 3 times with distilled water. The supernatant was concentrated and precipitated overnight at 4 °C. After centrifugation, the supernatant was removed and then re-dissolved in distilled water and deproteinated by the Savage method several times. The solution was precipitated again. After we obtained the crude polysaccharide by lyophilizing in the vacuum freeze dryer, the polysaccharide was passed through a Cellulose DEAE-52 column eluted by distilled water (800 mL). Subsequently, the column was eluted by NaCl solution with increasing concentrations (0.1–0.5 M) at a flow rate of 25 mL/h. The fractions were collected and detected by the phenol-sulfuric acid method [32]. The fractions were further purified by gel-filtration columns and obtained an acidic polysaccharide, defined as IAPS-2.

### 3.3. ELISA Analysis

The concentrations of IL-10, IL-12, VEGF, MMP-9, and IFN-γ in cell-culture supernatant or tumor tissues were measured by using the corresponding ELISA Kits (4A Biotech Co., Ltd., Beijing, China). Assays were performed according to the manufacturer’s protocol and read at 450 nm by using a microplate reader (Thermo Scientific, Waltham, MA, USA).

### 3.4. Quantitative Real-Time PCR

The total RNA was extracted from the cells using TRIzol^®^ Reagent (Invitrogen, Carlsbad, CA, USA) according to the manufacture’s instruction. The total RNA was quantified by using an Eppendorf Biophotometer Plus (Eppendorf AG, Hamburg, Germany). Then, a quantitative real-time qPCR was operated using LightCycler FastStar DNA Master SYBR Green I (Roche Diagnostics, Mannheim, Germany) following the instructions from the manufacturer. The fold change of each gene was normalized to that of β-actin. The primer sequences used in this experiment are listed as follows (F: forward; R: reverse): iNOS (5’-CCAAGCCCTCACCTACTTCC-3’ (F); 5’-CTCTGAGGGCTGACACAAGG-3’ (R)); Arginase-1 (5’-CCAGAAGAATGGAAGAGTCAGTGT-3’ (F); 5’-GCAGATATGCAGGGAGTCACC-3’ (R)); Mouse MHC II (5’-CATCTGCTCACGAGGTCTGGA-3’ (F); 5’-TGGCACTGGAGTGGCAAATAG-3’ (R)); Mouse Ym1 (5’-AGAAGGGAGTTTCAAACCTGGT-3’ (F); 5’-GTCTTGCTCATGTGTGTAAGTGA-3’ (R)); β-actin (5’-TGCTGTCCCTGTATGCCTCT-3’ (F); 5’-TTTGATGTCACGCACGATTT-3’ (R)).

### 3.5. Western Blotting Assay

The cellular proteins were extracted by lysing cells in a RIPA buffer containing 1mM Phenylmethylsulfonyl Fluoride (PMSF). The protein samples were separated by SDS-PAGE electrophoresis, and then transferred to PVDF membranes (Bio-Rad, Hercules, CA, USA). After blocking with bovine serum albumin (5%) for 1 h at room temperature under slight shaking, the membranes were incubated with primary antibodies (1:500) at 4 °C for 12 h and then blotted with secondary antibody for 2 h at room temperature. Then the bands were stained with SuperSignal West Pico Chemiluminescent Substrate (Thermo Scientific, USA). In this study, the cells including M2-M and TAMs were treated with 50 μg/mL IAPS-2 for 24 h, then the expression of phosphorylated P65 (p-P65), total P65, phosphorylated STAT1 (p-STAT2), total STAT1 (T-STAT1), phosphorylated STAT3 (p-STAT3) and total STAT3 (T-STAT3) in both cells were evaluated by Western blot. The corresponding antibodies, including phosphor-P65, Total-P65, phosphor-STAT1, Total-STAT1, phosphor-STAT3, Total-STAT3 and GAPDH were purchased from Cell Signaling Technology (Danvers, MA, USA).

### 3.6. The Effects of IAPS-2 on Macrophages and TAMs

Murine RAW 264.7 cells were first induced into M2 phenotype with 20 ng/mL IL-4, and then were treated with IAPS-2 (50 μg/mL) for 24 h. In addition, isolated TAMs were also stimulated with the same concentration of IAPS-2 for 24 h. Afterwards, the concentrations of IL-12 and IL-10 in cell-culture supernatant were measured using the corresponding ELISA kits (4A Biotech Co., Ltd., Beijing, China). The performance was strictly performed following the instructions.

### 3.7. Tumor Models

C57BL/6J mice, female, 6–8 weeks old, were provided by the Experimental Animal Centre of Nanjing Medical University (20180730, Nanjing, China). All mice were treated in strict accordance with the Nanjing University guidelines (Permit NO. 2011-039) and approved by the Animal Ethics Committee of Nanjing University. The mice were prepared for generating the heterotopic tumor model. Briefly, mouse sarcoma cells (S180 cells, 1 × 10^6^) were injected subcutaneously into the right armpit of the mice. The mice bearing tumors of similar sizes (the average tumor volume was about 50 mm) were selected for further study. IAPS-2 polysaccharide in PBS solution was administrated by intraperitoneal injection. We measured the tumor size with a caliper and weighed the tumor samples after harvesting. In another experiment, tumor cells were mixed with TAMs (2.5:1) treated with or without IAPS-2 and then were injected into the mice. Tumor diameters were measured every four days, and the tumor tissues were weighed.

### 3.8. In Vivo Anti-Tumor Effects of IAPS-2

The above mice were selected and randomly divided into different groups: Group I (PBS), Group II (control) and Group III (IAPS-2 at 50 mg/kg, ip). After treatment, the tumors in different groups were collected, and then tumor samples were fixed in Bouin’s buffer, 4% paraformaldehyde solution for 24 h; then, the tissues were embedded in paraffin and cut into slides at 5 μm. For histologic analyses, the sections were stained with hematoxylin and eosin (H&E). Each staining was repeated three times. For the immunofluorescence analysis the sections were washed with PBS 3 times and blocked with 5% BSA for 45 min and incubated with the corresponding primary antibodies including rabbit anti-mouse interleukin-10 (IL-10), rabbit anti-mouse interleukin-12 (IL-12), and hamster anti-mouse interferongamma (IFN-γ) at 4 °C for 12 h. After washing with tris-buffered saline 3 times, the sections were subsequently stained with the secondary antibodies: Alexa 488 labelled donkey anti-hamster, Alexa 546 labelled donkey anti-rabbit at room temperature for 2 h. Then, the sections were stained, with the nuclei being counterstained with diamidino-2-phenylindole (DAPI) at room temperature for 5 min. Finally, the sections were photographed using a confocal microscope (Nikon, Tokyo, Japan). Primary antibodies against IL-10, IL-12 were purchased from 4A Biotech Co., Ltd. (Beijing, China). Antibodies against IFN-ϒ were purchased from Biolegend (San Diego, CA, USA). Alexa 488 labelled donkey anti-hamster, Alexa 546 labelled donkey anti-rabbit were obtained from Life Technologies (Carlsbad, CA, USA).

### 3.9. Statistics

The results are presented as the means ± standard deviation (SD) of at least 3 independent experiments, and each dosage or treatment was tested in triplicate. The statistical analyses were performed using one-way ANOVA (GraphPad Prism 6, GraphPad Software Inc., San Diego, CA, USA), with ^#^ and ^##^ standing for *p* < 0.05 and *p* < 0.01, respectively.

## 4. Conclusions

In our study, a novel polysaccharide named IAPS-2, showed the significant anti-tumor effect in vivo. It exerted the anti-tumor effect through the NF-κB, STAT1 and STAT3 signal pathways, which could re-direct the type of TAMs both in vitro and in vivo. As such, IAPS-2 could modulate the function of TAMs and regain immune surveillance against tumors. This prospective finding further illustrated the mechanism of anti-tumor effects from herbal polysaccharide. It may become a valuable candidate for the development of a new macromolecule for cancer immunotherapy.

## Abbreviations

IAPS-2	*Ilex asprella* polysaccharide-2
TAM	Tumor-associated macrophages
M2-M	M2 type macrophages
